# Extending Standards for Genomics and Metagenomics Data: A Research Coordination Network for the Genomic Standards Consortium (RCN4GSC)

**DOI:** 10.4056/sigs.26218

**Published:** 2009-07-20

**Authors:** John C. Wooley, Dawn Field, Frank-Oliver Glöckner

**Affiliations:** 1 University of California San Diego, La Jolla California, USA; 2 NERC Center for Ecology and Hydrology, Oxford, United Kingdom; 3 Microbial Genomics Group, Max Planck Institute for Marine Microbiology and; Jacobs University Bremen, Bremen, Germany

## Abstract

Through a newly established Research Coordination Network for the Genomic Standards Consortium (RCN4GSC), the GSC will continue its leadership in establishing and integrating genomic standards through community-based efforts. These efforts, undertaken in the context of genomic and metagenomic research aim to ensure the electronic capture of all genomic data and to facilitate the achievement of a community consensus around collecting and managing relevant contextual information connected to the sequence data. The GSC operates as an open, inclusive organization, welcoming inspired biologists with a commitment to community service. Within the collaborative framework of the ongoing, international activities of the GSC, the RCN will expand the range of research domains engaged in these standardization efforts and sustain scientific networking to encourage active participation by the broader community. The RCN4GSC, funded for five years by the US National Science Foundation, will primarily support outcome-focused working meetings and the exchange of early-career scientists between GSC research groups in order to advance key standards contributions such as GCDML. Focusing on the timely delivery of the extant GSC core projects, the RCN will also extend the pioneering efforts of the GSC to engage researchers active in developing ecological, environmental and biodiversity data standards. As the initial goals of the GSC are increasingly achieved, promoting the comprehensive use of effective standards will be essential to ensure the effective use of sequence and associated data, to provide access for all biologists to all of the information, and to create interdisciplinary opportunities for discovery. The RCN will facilitate these implementation activities through participation in major scientific conferences and presentations on scientific advances enabled by community usage of genomic standards.

## Introduction

The sustained, rapid completion of whole genome sequences has transformed biological research for the entire breadth of scales from molecular to population and ecosystem level studies. Ensuring effective use of genomic and metagenomic data requires a community-driven process that initially establishes standardized mechanisms supporting acquisition, electronic capture and communication of all genomic and metagenomic data and subsequently, that enables the willingness and commitment for full participation in their usage. Implementation of such goals is also essential to ensure effective use of these data and equitable access by all biologists to all of this information; in turn, the availability of comprehensive, standards-driven information resources will create interdisciplinary opportunities for discovery.

The Genomic Standards Consortium (GSC) pioneered and has promoted the establishment of such community based, consensus building efforts. To engage the entire community, deliver high quality products and enable experimentalists and information resource providers to appreciate the value of consistent usage of the standards, GSC operates as an open organization. To build upon the initial successes, expand its international participation, and include additional research communities, the Genomic Standards Consortium (GSC) has established a Research Coordination Network (RCN) under US National Science Foundation funding.

The RCN, termed the Research Coordination Network for the Genomic Standards Consortium (RCN4GSC), will further the work of the GSC in promoting and integrating standards describing genomic and metagenomic data sets within the international community. Research Coordination Network awards are a unique mechanism of the National Science Foundation to build community efforts that are of central importance for 21^st^ Century biology. The creation of this RCN for the GSC will ensure a worldwide, community driven process for establishing standardized mechanisms for the electronic capture of genomic data and for obtaining willingness to participate. Achieving this goal is essential to ensure effective use of the sequence and associated data, to provide access for all biologists to all of the information, and to create interdisciplinary opportunities for discovery. In the process of sustaining and extending the pioneering efforts of the GSC, the RCN will primarily provide five years of funding (2009-2013) to support small collaborative workshops aimed at advancing core objectives and processes for standards and the exchange of early-career scientists between GSC research groups. The exchanges will allow rapid progress on technical deliverables of the GSC such as GCDML.

## Organization

The RCN will be led through its Steering Committee by John C. Wooley, UCSD (PI); Dawn Field, CEH Oxford; Frank Oliver Glöckner, MPI-Bremen; George Garrity, MSU; Nikos Kyrpides, JGI; Karen Nelson, JCVI; Owen White, University of Maryland, as drawn from the GSC Board. Other RCN members responsible for leading core projects and activities include James Cole, MSU; Peter Dawyndt, University of Ghent; Renzo Kottmann, MPI*-*Bremen; Lynette Hirschman, MITRE; Victor Markowitz, LBNL; Inigo San Gil, UNM; and Lynn Schriml, University of Maryland. Other steering committee members and core leaders will be selected to cover the expanded efforts in ecological, environmental and biodiversity data.

## Goals of the RCN4GSC

Expanding and fully incorporating early consensus building activities and the institutional commitments inspired and implemented by the GSC, the creation of the RCN4GSC will ensure continued worldwide, community-driven capacity to establish standardized mechanisms for the electronic capture of genomic data and for obtaining willingness to participate. The RCN4GSC, using open access solutions and engaged with other international working bodies, will largely focus on a set of already defined GSC ‘core projects’ as well as provide the community with a forum in which to propose and develop future consensus-drive activities when appropriate. Specifically, as an arm of the GSC, this RCN will focus extending of the “Minimum Information about a (Meta) Genome Sequence” (MIGS/MIMS) specification and the GSC Genome Catalogue [1], the Genomic Contextual Data Markup Language (GCDML) [2] the Genomic Rosetta Stone [3], Habitat-Lite (based on the Environment Ontology) [4] and the *Standards in Genomic Sciences* eJournal [5] which will contain, among other types of articles, genome and metagenome notes as well as Standard Operating Procedures (SOPs) [6]. The RCN will also further extend the genomic standards effort to engage the developers of data standards for ecological, environmental and biodiversity data.

Such efforts will work to ensure that extensive analyses can readily be done on all genome and metagenome data sets. Implementing this RCN will allow the standardization effort to partner closely with an inclusive set of major stakeholders and above all, with the community as a whole, to lead to the best product and ensure community support. This RCN, thus, addresses an important goal of 21^st^ century biology; namely, establishing an integrative biology across the vast scales of time, space and complexity of life.

## Promotion of Inclusive Participation

The GSC has always been an open organization and through this RCN, hopes to widen international participation in its core activities. The GSC and its RCN reflect the democratization of access, provided in principle by the internet, that can be enabled by the establishment of and compliance to standards that ensure all genomic data is available to the vast diversity of biologists. Thus, biologists with enthusiasm and a commitment to community service are always welcome to join and encouraged to participate actively.

Coordination with professional societies and communication to federal and foundation stake-holders will be among strategies for expanding participation, as will increasing participation from Asia. Similarly, the RCN will allow the GSC members to expand community networking to enlist new participants and inform federal and foundation stakeholders, as well as to reach more experimentalists through numerous contributions at major meetings. The contributions will focus on attributes like standards for metagenomics, metadata and meta-analysis, including brief updates and presentations of specific research advances enabled by the use of the standards. These venues for expanding participation will be managed by the Communications Committee, who will focus on enlisting further diversity in participation and in reaching out to beginning biologists. We will also ramp up efforts to coordinate with large sequencing centers and knowledge resources, both of which are represented on the RCN to share their experiences and sustain a dialogue around ongoing advances.

## Major Networking Activities

The RCN will sustain international GSC meetings and small group focused working meetings. Intense collaborations around the maturation of GCDML, the Genome Rosetta Stone and the Genome Catalogue will be an early focus. Productivity will be sustained year round by routine audio-video and web-enabled interactions, including small working group sessions and collaborations via the RCN's extant web hub at http://gensc.org. Experts in both genomics and computing will collaborate with domain specialists to extend GCDML and be sure its development is consistent with biological science expectations in general. These activities will also expand ongoing coordination required for implementation of all the GSC’s core projects, of all the GSC’s core projects, in particular in the context of the international standards community [7,8]. At the same time, the RCN will help the GSC to sustain and build new interactions outside the domain of molecular research. An important early activity will be further development of a two-way exchange between the NSF’s Long Term Ecological Research (LTER) Network and the GSC [9]. Other exchanges, including those with environmental and biodiversity data efforts as well as specific microbial and metagenomic efforts, will follow. It is critical that the GSC’s efforts evolve in close association with efforts to develop ecological data standards, such as Ecological Metadata Language (EML), biodiversity standards, such as Darwin Core, and environmental research programs, such as the Global Lake Ecological Observatory Network. For effective use of funds, smaller working group sessions will also occur at major professional society meetings when possible. The “e science” nature of this RCN also means that considerable progress can be made throughout the year via document sharing on wiki and blog environments for community input and continuous dialogue.
